# AI Chatbot Service Adoption Among C2C Second-Hand Sellers: An Integrated UTAUT-TTAT Framework

**DOI:** 10.3390/bs16091670

**Published:** 2026-09-17

**Authors:** Yurou Zhao, Xiao Yang, Kim-Shyan Fam

**Affiliations:** 1 Business School, Qingdao University of Technology, Qingdao 266520, China; yurouzhao@163.com; 2 School of Business and Management, Jilin University, Changchun 130012, China; 3 School of Management and Marketing, Romanian American University, 012101 Bucharest, Romania

**Keywords:** AI chatbots, C2C second-hand platforms, Unified Theory of Acceptance and Use of Technology (UTAUT), Technology Threat Avoidance Theory (TTAT), perceived effortlessness, perceived risk, adoption intention, dual mediation

## Abstract

Against the background of AI penetration in C2C second-hand commerce, AI chatbots have emerged as vital operational tools for individual sellers. Prior technology adoption literature predominantly emphasizes positive acceptance drivers yet overlooks the simultaneous existence of benefit and risk perceptions, and cannot fully explain individual sellers’ paradoxical adoption intentions. This study integrates the Unified Theory of Acceptance and Use of Technology (UTAUT) and Technology Threat Avoidance Theory (TTAT) to construct an integrated dual mediation model, adopting perceived effortlessness and perceived risk as parallel mediators. Online questionnaire surveys were distributed to individual sellers operating on Chinese C2C second-hand trading platforms. After screening and data cleaning, a final valid sample of 261 respondents was retained. Partial least-squares structural equation modelling (PLS-SEM) was employed to empirically test the proposed research model. The results demonstrate that personal factors (perceived busyness, desire for control, AI acceptance) and situational factors (product standardization, social influence, platform safeguards) jointly shape individual sellers’ dual perceptions. These antecedents exert differentiated impacts on the two mediating constructs, which subsequently predict individual sellers’ intentions to adopt AI chatbots. This research clarifies the balancing mechanism of positive and negative perceptions in technology decision-making. The framework reconciles conflicting psychological evaluations of intelligent systems, deepens the understanding of paradoxical adoption behaviors, and offers practical implications for optimizing intelligent services within C2C second-hand ecosystems.

## 1. Introduction

Driven by the expansion of global e-commerce and the booming circular economy, consumer-to-consumer (C2C) second-hand trading platforms have emerged as a vital segment of the digital business ecosystem (Korhonen et al., 2018; S. F. Liu & Wang, 2025). As C2C resale models gain widespread popularity worldwide, the scale of the global second-hand e-commerce market was estimated to reach USD 36.5 billion in 2025. The proliferation of online trading channels has greatly enabled individual sellers to participate in idle commodity transactions. However, individual platform operators generally face inherent operational challenges, including intensive repetitive customer consultations, fragmented working time, and heavy daily operational workloads.

To mitigate individual sellers’ communication pressures and improve transaction efficiency, AI chatbots have been widely adopted across various e-commerce scenarios. Practical evidence and academic research have demonstrated that AI chatbots can effectively shorten response time, reduce manual communication costs, and boost transaction conversion rates (Fan et al., 2023; Iddris et al., 2026). Nevertheless, unlike standardized business-to-consumer (B2C) e-commerce transactions, C2C second-hand trading scenarios feature non-standardized products, trust-dependent interactions, and personalized communication demands (Peng et al., 2023). This distinction leads to individual sellers’ ambivalent attitudes toward AI chatbots. While individual sellers intend to adopt such tools to reduce workloads and streamline operations, they also worry about potential risks such as inaccurate algorithmic replies, inconsistent communication styles, impaired buyer trust and loss of conversational control. These conflicting perceptions form a typical adoption paradox: individual sellers have strong functional demands for AI chatbots, yet their actual adoption willingness remains low.

This prevalent practical paradox raises a core research concern: What determinants shape individual sellers’ behavioral intention to adopt AI chatbots on C2C second-hand platforms?

A systematic literature review identifies three critical research gaps. First, existing scholarship on AI chatbot adoption predominantly focuses on standardized B2C scenarios, with limited attention to C2C second-hand markets featuring non-standardized goods and trust-based transactions (Fan et al., 2023). Second, most studies adopt consumer or firm-level perspectives, while individual sellers’ adoption decisions remain underexplored (Jiang et al., 2022; Q. Chen et al., 2023; Myin & Watchravesringkan, 2024). Third, mainstream acceptance theories emphasize positive utility perceptions yet overlook risk-induced avoidance tendencies (Sharma et al., 2025). Although some studies have combined UTAUT with risk-related constructs or TAM, as exemplified by Tian et al. (2025), such research mostly treats risk as an externally added variable rather than a parallel psychological mechanism alongside benefit perceptions, which weakens their ability to explain individual sellers’ trade-offs between efficiency benefits and uncertainties. More importantly, compared with B2C commerce, C2C second-hand transactions are characterized by heterogeneous product standardization, informal trust-based interactions, and limited platform institutional safeguards. These features amplify the tension between benefit-seeking and risk-avoidance among individual sellers within this setting, rendering it an ideal context for unpacking the aforementioned adoption paradox.

To address these theoretical limitations, contemporary behavioral and information system studies have called for integrated dual-path frameworks that combine positive facilitation and negative inhibition mechanisms (Cao et al., 2021). In the context of C2C second-hand transactions, individual sellers’ AI adoption intention is governed by two competing psychological pathways. The positive pathway is dominated by perceived effortlessness, which derives from the time-saving and workload-reducing benefits brought by AI intelligent services. Conversely, the negative pathway is constrained by perceived risk, which arises from individual sellers’ concerns about technological instability and transactional uncertainty. Importantly, these two pivotal perceptual states are not formed in isolation but are jointly shaped by individual sellers’ inherent personal traits (e.g., perceived busyness, desire for control) and external platform situational conditions (e.g., product standardization, platform safeguards, social influence). To date, few studies have incorporated personal factors, situational factors, perceived effortlessness, and perceived risk into a unified theoretical model to systematically unpack the underlying formation mechanism of individual sellers’ AI adoption intention in C2C second-hand contexts.

Accordingly, this study formulates its core research question: How do personal and situational factors shape individual sellers’ intention to adopt AI chatbots on C2C second-hand platforms via the dual mediating pathways of perceived effortlessness (positive mechanism) and perceived risk (negative mechanism)? This study integrates the unified theory of acceptance and use of technology and Technology Threat Avoidance Theory (TTAT) to elucidate sellers’ AI adoption intention through a dual-path framework covering both positive motivation and negative inhibition. Unlike conventional single-path acceptance models, this paradigm effectively explains sellers’ unique trade-offs between benefits and uncertainties in C2C second-hand transactions. Theoretically, this study extends intelligent tool adoption research to the under-examined circular e-commerce context and remedies the explanatory limitations of single-dimensional theoretical perspectives. Practically, it provides actionable insights for platforms to optimize AI services and facilitate sellers’ intelligent operational transformation.

## 2. Literature Review

### 2.1. AI Chatbots in E-Commerce

Fueled by continuous advances in natural language processing and machine learning, AI chatbots have become essential infrastructure within modern digital commerce ecosystems (Zhang et al., 2024). Widely implemented across e-commerce contexts, they undertake transaction consultation, bargaining, order management and after-sales dispute handling. Unlike traditional manual customer service restricted by fixed working hours, delayed replies, repetitive workflows and high labour costs, AI systems deliver round-the-clock service, automatic dialogue processing and standardized service outputs. Such technical merits reduce operational friction and ease the supply–demand imbalance in online communication services (X. Wang et al., 2022).

Existing studies on e-commerce AI services fall into three major categories: technical efficiency, user experience and technology adoption. In terms of technical performance, empirical evidence confirms that AI customer service shortens response time, cuts operational costs and improves conversion rates, thus optimising digital service systems (X. Wang et al., 2022). From the user experience perspective, scholars explore how AI attributes including response accuracy, interaction fluency and anthropomorphism shape user trust, satisfaction and continuous usage intention (Nicolescu & Tudorache, 2022; J. Li et al., 2023). Adoption research on AI chatbots frequently draws on classical frameworks such as the Technology Acceptance Model (TAM) and the Unified Theory of Acceptance and Use of Technology (UTAUT), verifying that perceived usefulness and perceived ease of use predict technology acceptance (M. Z. Liu et al., 2024; G. Chen et al., 2025).

Despite substantial progress, relevant research is largely situated in B2C e-commerce and centers on brand merchants and consumers (Stoilova, 2021). Prior AI chatbot research often conceptualizes chatbots as generic information technology rather than interactive conversational agents. Unlike traditional static information systems, chatbots rely on real-time dialogue and may simultaneously deliver efficiency gains and communication risks (Adam et al., 2021). Most existing empirical work focuses on consumers’ trust and satisfaction, whereas seller-side adoption decisions remain underexplored. The contextual disparities between standardized B2C transactions and C2C second-hand trading imply that established findings may not be readily generalizable. For individual sellers, chatbot adoption involves a unique trade-off between operational cost savings and reputational risks, which is rarely examined in C2C second-hand contexts.

### 2.2. Behavioral and Decision Characteristics of Individual Sellers on C2C Second-Hand Platforms

As an important pillar of the circular economy, C2C second-hand e-commerce has expanded rapidly thanks to low entry barriers and decentralised operations. Distinct from formal B2C merchants with standardised workflows and dedicated service teams, C2C platforms are dominated by individual sellers lacking systematic operational resources and professional customer service support (L. Wang & Sun, 2023). These independent operators generally have fragmented schedules, limited operational capacity and low risk tolerance, forming unique patterns of technology adoption.

Second-hand transactions feature highly non-standardised goods, inconsistent item conditions, ambiguous pricing and uncertain transaction outcomes. Consequently, C2C trading relies heavily on personalised buyer–seller interactions and interpersonal trust, generating greater communication costs and transaction uncertainty than conventional e-commerce (Moriuchi & Takahashi, 2022; Jang & Kim, 2023). Individual sellers must allocate scattered time to repeated enquiries, price negotiations and after-sales conflicts, resulting in heavy daily operational burdens. Furthermore, without brand endorsement and stable commercial systems, individual sellers prioritise low-cost, low-risk and lightweight operations. They are therefore more sensitive to technological risks while having strong practical demands for labour-saving intelligent tools.

Prior AI adoption literature predominantly focuses on consumer users or firm-level B2C AI deployment, leaving C2C second-hand individual sellers largely understudied (Fan et al., 2023). These individual sellers exhibit ambivalent attitudes toward AI chatbots: they pursue intelligent tools to reduce operational burdens while remaining cautious of technological and transactional risks. Although existing C2C marketplace studies have well documented interpersonal trust and platform governance as core strategies to mitigate information asymmetry and uncertainty, most relevant investigations concentrate on buyer trust formation and purchase decisions (Pavlou & Gefen, 2004). Systematic understanding of how platform institutional mechanisms shape individual sellers’ risk perceptions and subsequent AI adoption behaviors remains limited, creating an unaddressed research gap in second-hand commerce AI studies.

### 2.3. Theories of Digital Technology Acceptance and Avoidance

To resolve users’ ambivalent technology adoption behaviors in digital transaction contexts, recent e-commerce and technology behavior scholarship has increasingly integrated acceptance and avoidance theoretical lenses to capture both benefit-seeking and risk-avoidance mechanisms (Cao et al., 2021; Carpenter et al., 2019). While traditional acceptance theories fall short of explaining contradictory adoption decisions, such combined frameworks help unpack mixed attitudes toward emerging intelligent technologies. Nevertheless, most existing integrative studies focus on general consumer scenarios, with limited theoretical and empirical evidence regarding AI chatbot adoption among individual C2C second-hand sellers.

Research on individual technology adoption in online commerce has yielded a rich set of theoretical frameworks for understanding user responses to new digital services. The Unified Theory of Acceptance and Use of Technology (UTAUT) represents a dominant framework for explaining individual technology acceptance behavior, positing that favourable cognitive perceptions of new technologies promote usage intentions (Venkatesh et al., 2003). Nevertheless, conventional UTAUT constructs focusing on general usefulness and ease-of-use fail to capture the fragmented, lightweight, and low-cost operational realities facing informal individual sellers within C2C second-hand trading environments.

Against this backdrop, this study introduces perceived effortlessness to evaluate technology decision-making in the second-hand e-commerce context. Perceived effortlessness refers to users’ subjective belief that technological tools save time, reduce energy expenditure and streamline workflows (S. M. Sohn, 2025). For C2C second-hand individual sellers without professional service teams and sufficient time resources, AI chatbots are adopted primarily to replace repetitive manual communication and lower redundant interaction costs rather than expand business scale or raise profits. Existing evidence indicates that intelligent tools offering high effortlessness improve user experience and adoption willingness (Lopes et al., 2025). Accordingly, perceived effortlessness acts as a vital positive mediator shaping individual sellers’ intention to adopt AI chatbots.

To complement acceptance-oriented perspectives, Technology Threat Avoidance Theory (TTAT) provides a lens to analyse decision-making under high transaction uncertainty (Carpenter et al., 2019). Its central construct, perceived threat, captures users’ subjective evaluation of potential instability and negative consequences brought by intelligent technologies. When individuals anticipate technological hazards, they develop hesitation and avoidance tendencies (Liang & Xue, 2009), offsetting the tendency of traditional adoption models to overemphasize functional benefits while overlooking risk resistance. Aligned with C2C second-hand trading characteristics, this study extends perceived threat into perceived risk, encompassing worries over AI response errors, transaction failure, eroded trust and reputational damage in second-hand transactions.

Intrinsic features of C2C second-hand trading amplify unique risks associated with AI chatbot application. Inappropriate automated replies arising from non-standard products and personalised communication may trigger buyer dissatisfaction and transaction losses. In addition, individual sellers value control over interpersonal exchanges and flexible transaction arrangements; over-reliance on AI may undermine such personalized trading advantages, and resultant risks will suppress adoption intentions. Prior work confirms that personal traits and situational factors jointly determine individual risk perceptions in online trading scenarios (Slovic, 1987; Silva et al., 2023). Even so, the literature lacks systematic analysis of the antecedents and outcomes of seller-side risk perceptions within the context of AI services on C2C second-hand platforms. Few studies adequately explain individual sellers’ ambivalent adoption behaviors driven simultaneously by efficiency aspirations and risk concerns in second-hand e-commerce.

Building on the above discussion, integrating UTAUT and TTAT offers a theoretically robust way to address these gaps (Cao et al., 2021). UTAUT illuminates adoption motivations rooted in functional and social drivers, yet it neglects transaction uncertainty, threat perception and avoidance psychology. TTAT, by contrast, focuses on risk assessment and technology resistance but cannot capture efficiency-driven adoption incentives. This integrated dual-path framework simultaneously incorporates effort-saving motives and risk concerns, enabling a holistic explanation of C2C sellers’ ambivalent AI adoption decisions that cannot be fully captured by either theory alone.

## 3. Research Model and Hypotheses

### 3.1. Conceptual Research Model

**Figure 1:**
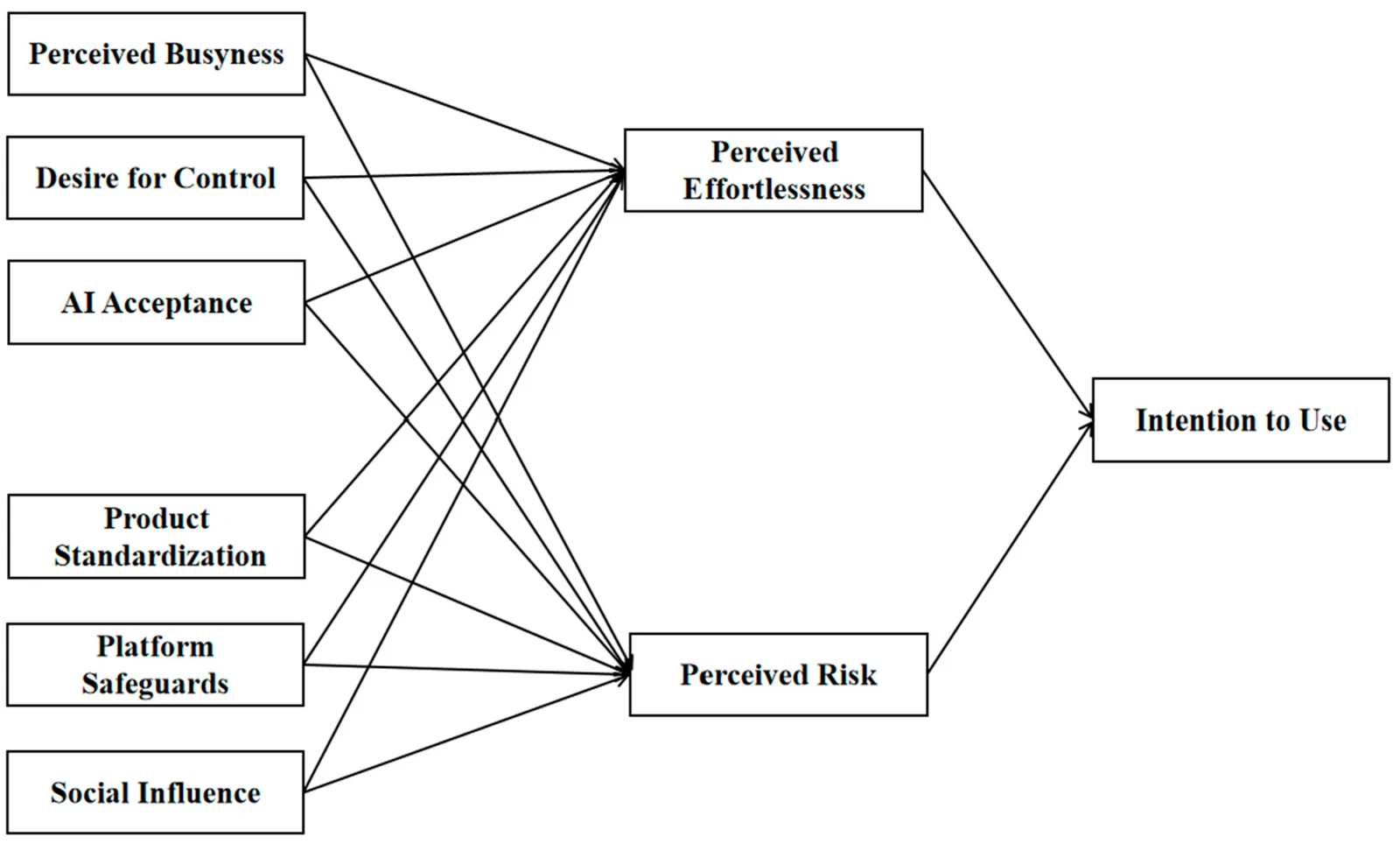
Proposed Theoretical Model of AI Chatbot Use Intention among C2C Individual Sellers.

To achieve the research aim, we develop and test a theoretical model (see Figure 1) to understand the factors and their effects on individual sellers’ attitudes and behavioral intentions to use AI chatbots on C2C second-hand platforms.

As elaborated in the previous chapters, AI chatbots generate both positive values and potential concerns for individual sellers on C2C second-hand platforms. Nevertheless, existing literature mainly adopts unilateral theoretical perspectives and fails to construct integrated models that simultaneously account for both benefits and risks (Breward et al., 2017). Although UTAUT has been very widely used to predict the behavioral intention towards technology acceptance, it does not consider the effect of negative perceptions and concerns of users and their impact on the users’ behavioral intentions. Therefore, its power to predict users’ attitudes and intentions could be limited in the context of AI-hosted services in C2C second-hand transactions. Recently, researchers also called for the development of integrated models that combine both acceptance and avoidance mechanisms to explain contradictory technology adoption behaviors (Cao et al., 2021).

Drawing on the unique characteristics of C2C individual sellers and second-hand transactions, this study adopts the person–environment fit perspective to select and categorize six antecedents into personal and situational factors. Personal factors reflect individual sellers’ inherent psychological traits and subjective perceptions, including perceived busyness, desire for control, and AI acceptance. Situational factors represent external transactional and platform conditions in C2C second-hand markets, consisting of product standardization, social influence, and platform safeguards. These six antecedents are theoretically grounded in the integrated logics of UTAUT and TTAT. UTAUT emphasizes functional and social drivers that shape users’ benefit-oriented technology evaluations, underpinning the formation of perceived effortlessness. In contrast, TTAT focuses on threat appraisal and risk perception, laying the theoretical foundation for perceived risk. Regarding personal factors, perceived busyness and AI acceptance align with UTAUT’s utility-oriented logic and primarily enhance perceived effortlessness, whereas desire for control corresponds to TTAT’s threat-perception mechanism and increases perceived risk. For situational factors, product standardization and social influence originate from UTAUT’s contextual and social cues and positively affect perceived effortlessness, while platform safeguards follow TTAT’s risk-mitigation logic and reduce perceived risk. Collectively, these antecedents influence sellers’ adoption intention through two parallel mediating pathways. We adopt parallel rather than sequential mediation. Perceived effortlessness and perceived risk are independent, concurrent appraisals with distinct dimensions; sellers do not form one perception after the other. Rooted in UTAUT and TTAT, our model aims to test antecedents’ competing indirect effects via these two separate pathways, making parallel mediation more theoretically appropriate.

In summary, the research model proposed in this study is based on three core considerations derived from the unique context of AI chatbot adoption among individual sellers on C2C second-hand platforms:

- Incorporation of situational factors. C2C second-hand transactions are restricted by objective external environments such as product attributes, peer interactions and platform rules. These situational conditions act as important driving forces and constraints for individual sellers’ perception and behavior. Accordingly, this model incorporates typical situational factors as antecedent variables, to reflect the influence of real transaction scenarios on AI service adoption.
- Human-centered approach to technology adoption. Studying individual sellers’ behavioral intentions toward using AI chatbots must follow a human-centered perspective. Individual sellers’ inherent personal traits such as work status, psychological tendencies and technology attitudes fundamentally shape their perceptions of intelligent tools. Therefore, the research model takes various personal factors as key antecedent variables, to explore how individual characteristics drive subsequent behavioral decisions.
- Inclusion of both technology acceptance and avoidance factors. AI chatbot adoption in C2C second-hand trading contexts has the potential to generate both positive and negative impacts on individual sellers’ operational experiences. To interpret individual sellers’ mixed attitudes toward AI tools, the model integrates two parallel mediating mechanisms: perceived effortlessness representing positive adoption motivation and perceived risk representing negative avoidance psychology. This dual-path design can fully explain individual sellers’ contradictory decision-making logic.

All constructs within the theoretical framework are contextualized for C2C second-hand trading, and the full research framework is illustrated in Figure 1. Based on UTAUT, TTAT and existing literature, this study develops a set of research hypotheses to examine the linkages among situational factors, personal factors, two parallel mediating constructs and individual sellers’ AI chatbot use intention.

### 3.2. Development of Research Hypotheses

#### 3.2.1. Personal Antecedent Factors

Perceived busyness refers to individuals’ subjective feeling of heavy workload and task occupancy derived from their judgment of daily schedules (Kim et al., 2019). It essentially represents cognitive resource scarcity resulting from time limits. Individual sellers in decentralized second-hand transactions face frequent repetitive inquiries, which continuously drain cognitive resources and raise operational burdens. According to cognitive resource theory, human cognitive capacity is limited; individuals with scarce cognitive resources prefer options that conserve mental resources (Kahneman, 1973). For individual sellers, AI customer service effectively cuts workload. Hence, individual sellers with higher perceived busyness value the effort-saving utility of AI customer service more, meaning perceived busyness positively affects perceived effortlessness. Meanwhile, individual sellers exhaust limited cognitive resources in assessing the tool’s effortless advantages, leaving little mental capacity to recognize hidden risks of AI customer service and lowering their perceived risk. Accordingly, perceived busyness negatively influences perceived risk. Based on the above reasoning, this paper proposes the following hypotheses:

**H1a.**  *Perceived busyness has a positive effect on perceived effortlessness*.

**H1b.**  *Perceived busyness has a negative effect on perceived risk*.

Desire for control is a stable personality trait reflecting people’s tendency to dominate events, social interactions and decision outcomes (Burger, 2013). In online transactions, users’ control over communication and processes shapes their experience and risk judgment. When evaluating perceived effortlessness, individual sellers with high desire for control prioritize communication autonomy. Standardized AI customer service templates restrict personalized expression and break natural dialogue. Although AI reduces repetitive typing, high-control individual sellers feel constrained and incur extra psychological costs, weakening their recognition of AI’s effort-saving value. Thus, desire for control negatively predicts perceived effortlessness. From perceived risk theory, losing control causes discomfort as users cannot anticipate dialogue and service results (Faraji-Rad et al., 2017). Individual sellers worry rigid automated replies trigger misunderstandings, buyer complaints and transaction disputes, raising perceived risk. The convenience of AI cannot offset these concerns. Therefore, desire for control positively predicts individual sellers’ perceived risk. Based on the above reasoning, this paper proposes the following hypotheses:

**H2a.**  *Desire for control has a negative effect on perceived effortlessness*.

**H2b.**  *Desire for control has a positive effect on perceived risk*.

AI acceptance represents individuals’ overall attitude, recognition and behavioral inclination toward artificial intelligence technologies. Rooted in the classic technology acceptance framework, users’ general acceptance of emerging technologies fundamentally shapes their subsequent cognitive evaluation and usage decisions (Gursoy et al., 2019). It is worth noting that AI acceptance differs from AI chatbot use intention. AI acceptance reflects general attitudes toward AI technology, while use intention describes sellers’ specific willingness to adopt AI chatbots for second-hand selling. Individual sellers with higher AI acceptance are more likely to recognize the advantages of AI services in simplifying workflows and cutting operational costs, which in turn enhances their perceived effortlessness. Meanwhile, positive attitudes toward AI can alleviate users’ inherent doubts about algorithm stability, service rationality and technical failure, thereby reducing perceived risk (K. Sohn & Kwon, 2020). Therefore, AI acceptance is assumed to positively influence perceived effortlessness and negatively influence perceived risk.

**H3a.**  *AI acceptance has a positive effect on perceived effortlessness*.

**H3b.**  *AI acceptance has a negative effect on perceived risk*.

#### 3.2.2. Situational Antecedent Factors

Product standardization refers to the uniformity of product specifications, physical conditions and description criteria on second-hand platforms. From the perspective of transaction cost theory, unified standards can effectively reduce information asymmetry and repetitive communication costs between transaction parties (Y. Li & Zhang, 2024). For individual sellers adopting AI chatbots, highly standardized products reduce repeated explanatory work, ease the pressure to supplement information through conversations, greatly relieve operational burdens and boost perceived effortlessness. In addition, consistent product norms reduce the probability of discrepancies in product status, communication barriers induced by rigid AI automated replies, buyer–seller disputes and post-transaction conflicts, thereby mitigating individual sellers’ perceived transaction risk. We therefore propose the following hypotheses:

**H4a.**  *Product standardization has a positive effect on perceived effortlessness*.

**H4b.**  *Product standardization has a negative effect on perceived risk*.

Platform safeguards include transaction protection rules, after-sales management mechanisms and dispute resolution services provided by the platform. As formal institutional guarantees for online transactions, platform support systems can share individual sellers’ operational risks and cut extra mental energy spent on negotiations and rights defense (Puntoni et al., 2021). Specifically, sound platform safeguards simplify daily operations and boost individual sellers’ perceived effortlessness. Meanwhile, official platforms guarantee hedge various potential risks stemming from AI chatbots and online transactions, which effectively reduce individual sellers’ perceived risk. The corresponding hypotheses are proposed as follows:

**H5a.**  *Platform safeguards have a positive effect on perceived effortlessness*.

**H5b.**  *Platform safeguards have a negative effect on perceived risk*.

Social influence reflects the pressure from important referents who encourage or persuade individual sellers to adopt AI chatbots (Venkatesh et al., 2003). When individual sellers receive positive opinions and practical experience sharing from peers, friends or platform influencers, they tend to form optimistic expectations regarding the functionality of AI tools. Such social cues help individual sellers recognize the labour-saving advantages of AI chatbots and reduce their hesitation to accept the effort-saving value brought by intelligent services. Accordingly, stronger social influence facilitates higher perceived effortlessness. Conversely, insufficient or negative social information will amplify uncertainty. If most referents express worries about automated AI replies, transaction conflicts and hidden operational risks, individual sellers will internalize these concerns. Even when AI tools objectively reduce repetitive work, adverse social signals make individual sellers more alert to potential pitfalls of AI application and raise their perceived risk. Taken together, this study proposes the following hypotheses:

**H6a.**  *Social influence has a positive effect on perceived effortlessness*.

**H6b.**  *Social influence has a negative effect on perceived risk*.

#### 3.2.3. Mediating Mechanisms and Outcome Variable

Perceived effortlessness refers to the core functional benefit perception brought by AI chatbots for individual sellers. Based on the benefit-perception logic of technology adoption theory, users will generate stronger adoption motivation once they perceive that an information tool can cut repetitive labor and relieve daily operational burden (S. M. Sohn, 2025). Mass existing technology acceptance studies have verified that positive perceived functional benefits can significantly boost users’ behavioral intention to implement new digital tools (H. Li et al., 2026). When individual sellers feel that AI customer service can save communication energy and reduce repetitive reply work, their willingness to use such tools will rise accordingly. Therefore, this study proposes the following hypothesis:

**H7.**  *Perceived effortlessness has a positive effect on individual sellers’ intention to use AI chatbots*.

Perceived risk reflects individual sellers’ subjective concerns about potential adverse outcomes arising from AI chatbot services, including communication misunderstanding, transaction disputes and personal reputation damage (Marjerison et al., 2025). From the perspective of risk avoidance psychology, high perceived uncertainty will create obvious psychological resistance and usage barriers. If individual sellers anticipate that automated machine replies may trigger various service failures and economic losses, they will hold a more conservative attitude toward AI tools and restrain their adoption willingness. Accordingly, we put forward the following hypothesis:

**H8.**  *Perceived risk has a negative effect on individual sellers’ intention to use AI chatbots*.

Drawing on the above direct paths, this study further proposes a parallel mediating mechanism. All personal-level and situational antecedent factors can shape individual sellers’ adoption intention indirectly by altering their perceived effortlessness and perceived risk toward AI chatbots. The two perceptual states serve as core transmission channels linking multi-source predictors to behavioral willingness. Thus, the following hypotheses are proposed:

**H9.**  *Perceived effortlessness mediates the effects of perceived busyness, desire for control, AI acceptance, product standardization, platform safeguards, and social influence on individual sellers’ intention to use AI chatbots*.

**H10.**  *Perceived risk mediates the effects of perceived busyness, desire for control, AI acceptance, product standardization, platform safeguards, and social influence on individual sellers’ intention to use AI chatbots*.

## 4. Research Methodology

This study adopts a cross-sectional questionnaire survey to collect primary data from C2C second-hand individual sellers and employs partial least squares structural equation modeling (PLS-SEM) implemented via SmartPLS 4 to test the proposed model. This analytical approach is recommended for research contexts with underdeveloped theoretical frameworks (Hair et al., 2013; Cao et al., 2021). Although the UTAUT theoretical system is well-established and empirically validated, TTAT lacks sufficient empirical verification (Carpenter et al., 2019). This study further develops the first integrated framework combining UTAUT and TTAT specifically within the C2C retail context. Accordingly, PLS-SEM is deemed highly suitable for empirically testing the proposed model.

### 4.1. Measures of Constructs

All focal constructs were measured using seven-point Likert scales, ranging from 1 = strongly disagree to 7 = strongly agree. Measurement items were adapted from prior studies and revised to match the research context. A brief pretest was carried out prior to the formal survey to verify item clarity and contextual appropriateness. The three self-developed constructs were subjected to content validity assessment via expert review and pretest.

Perceived Busyness was measured using three items from prior studies (Martin & Park, 2003; Kim et al., 2019), which capture individual sellers’ persistent heavy operational workload in C2C second-hand transactions. Three items revised from Burger and Cooper (1979) were applied to measure desire for control, which reflects individual sellers’ psychological tendency to dominate buyer communication instead of relying on automated AI replies. AI Acceptance was measured using three items from Schepman and Rodway (2020), capturing individual sellers’ general positive predisposition toward AI service tools.

Product Standardization was measured using three self-developed items. Drawing on the conceptual logic of product homogeneity in commoditization research (Reimann et al., 2010), these items assess the degree of uniform listing information disclosure for respondents’ second-hand goods. Platform Safeguard was measured using three self-developed items. Drawing on the institutional assurance logic for online marketplaces (Pavlou & Gefen, 2004), these items capture individual sellers’ perceptions of the platform’s institutional safeguards for AI chatbot services, including operational guidelines, transaction security guarantees, and liability/compensation arrangements for AI-induced service errors. Social Influence was measured using three items adapted from prior studies (Venkatesh et al., 2012; Gansser & Reich, 2021), which capture individual sellers’ beliefs that people they consider important believe they ought to use AI chatbot services.

Perceived effortlessness was measured using three self-developed items based on prior studies (S. M. Sohn, 2025) regarding the extent to which AI helps a seller save time, reduce workload, and reduce repeated explanation. Perceived risk was measured with three items adapted from Marjerison et al. (2025), which assess individual sellers’ concerns over transaction disputes, inappropriate replies and information errors caused by AI customer service chatbots.

Three items from Venkatesh et al. (2012) were used to measure intention to use with reference to the extent to which a seller will use AI in the future, in transactions, and/or frequently.

Table 1 summarizes the focal constructs, abbreviations, number of items, and main source domains. Full item wording is provided in Supplementary Table S1.

### 4.2. Sample and Data Collection

The target respondents were C2C second-hand individual sellers on domestic idle trading platforms represented by Xianyu, who were aware of the platform’s AI automatic reply chatbot function. Questionnaires were distributed from April to May 2026 across Xianyu individual seller WeChat groups and second-hand trading online forums. Strict screening rules were set to filter out non-sellers and respondents unfamiliar with AI customer service tools. Questionnaires featuring identical single-point scoring or unreasonably short filling durations were eliminated to guarantee data quality. After multiple follow-ups, 280 questionnaire responses were received, of which 261 valid responses were retained for subsequent analysis, with an effective response rate of 93.2%.

### 4.3. Respondents

Table 2 presents the demographic and operational profiles of 261 valid respondents, including gender, age and years of second-hand operation. In terms of gender, male respondents accounted for 55.17% and female respondents accounted for 44.83%. For age distribution: 18–25 years old (24.14%), 25–35 years old (34.87%), 35–45 years old (29.50%) and above 45 years old (11.49%). In terms of operating years: less than 1 year (10.34%), 1–3 years (21.46%), 3–5 years (44.83%), above 5 years (23.37%). Regarding monthly transaction frequency: fewer than 5 orders per month (45.21%), 5–10 orders per month (32.95%), 10–20 orders per month (14.56%), more than 20 orders per month (7.28%). In terms of AI chatbot usage experience, 45.98% of individual sellers have used AI chatbots for second-hand selling, while 54.02% have never used such tools. Among AI chatbot users, respondents reported three self-reported usage levels: rarely (38.33%), occasionally (35.00%), and frequently (26.67%). The respondents trade a wide range of second-hand goods including apparel, digital gadgets and home supplies, which enhances the sample’s representativeness for the population of C2C individual sellers.

### 4.4. Common Method and Non-Response Bias

Procedural remedies were adopted to mitigate common method bias before formal data collection. Full anonymity of respondents was guaranteed in the survey cover letter to reduce the tendency to provide socially desirable responses. Item ambiguity was minimized by defining each indicator clearly and designing concise, specific questions. All measurement items were randomly mixed without labeling construct names or grouping items by latent variables, which restrained respondents from speculating and artificially matching hypothesized correlations. Positively and negatively worded items were mixed to control acquiescence and disacquiescence biases (Podsakoff et al., 2012).

For statistical examination of potential common method bias, the partial correlation procedure proposed by Lindell and Whitney (2001) was utilized. This approach relies on a marker variable that theoretically bears no relationship with the key constructs in the research model to compare zero-order correlations and partial correlations after controlling method variance. In this study, respondents’ operating years were selected as the marker variable. Consistent with landmark UTAUT studies, operating years have no inherent theoretical linkage with the UTAUT-based latent constructs in our model. The correlation matrix confirmed that operating years were not statistically correlated with the dependent variable Intention to Use or most of the independent constructs. The results of the partial correlation procedure revealed no meaningful shifts in the magnitude and significance of all pairwise correlations, which indicates that common method bias was not a serious concern in this research (Lindell & Whitney, 2001).

An independent sample *t*-test was conducted to assess non-response bias. We split the total sample into two subgroups: the first 30% of early responses and the last 30% of late responses collected via multiple follow-ups. The *t*-test results showed no significant between-group differences in demographic attributes and the mean scores of all latent constructs (*p* > 0.05), demonstrating that non-response bias would not distort the subsequent PLS-SEM analysis.

## 5. Model Testing and Findings

### 5.1. Evaluation of the Measurement Model and Structural Model

First, the measurement model was evaluated. To avoid measurement model misspecification, we conducted a confirmatory tetrad analysis (CTA-PLS), which confirmed that the measurement model was a reflective model. We evaluated the reflective measurement model by considering the internal consistency (composite reliability), indicator reliability, convergent validity and discriminant validity. The evaluation results were satisfactory as summarized in Table 3 and Table 4. Discriminant validity was also further established as the scores of the heterotrait-monotrait (HTMT) ratio of correlations were below the suggested threshold of 0.85 (Benitez et al., 2020) (see Table 5). 

Second, we assessed the structural model in terms of collinearity and the significance and relevance of the structural model relationships. The result of a bootstrapping procedure (5000 samples) indicated that no collinearity issue was present. The model’s predictive power was assessed by the amount of variance attributed to the latent variables (i.e., R^2^). The R^2^ values indicated that the full model explained 39.2% of the variance in intention to adopt AI chatbots, 42.5% in perceived effortlessness, and 38.7% in perceived risk. Following Wetzels et al. (2009), for PLS-SEM studies in information systems, an R^2^ greater than 0.36 indicates large explanatory power. The R^2^ value of the endogenous construct, intention to adopt AI chatbots, exceeds this cutoff, demonstrating the structural model has strong explanatory power. We further applied the blindfolding procedure to calculate q^2^ for evaluating out-of-sample predictive relevance. The q^2^ values for perceived effortlessness, perceived risk, and adoption intention were 0.275, 0.304, and 0.338, respectively, indicating moderate to substantial out-of-sample predictive relevance of the structural model.

### 5.2. Hypothesis Testing

#### 5.2.1. Direct Effects

We first examine the direct relationships proposed in the research model. We start with the personal antecedent factors. H1a proposes that perceived busyness has a positive effect on perceived effortlessness, which is supported as the effect is statistically significant, with the path coefficient of 0.351 (*p* < 0.001). H1b posits that perceived busyness has a negative influence on perceived risk, which is supported, with the path coefficient of −0.208 (*p* < 0.001). H2a predicts that desire for control negatively affects perceived effortlessness and is supported (β = −0.125, *p* = 0.019), while H2b, which argues desire for control positively influences perceived risk, receives support (β = 0.172, *p* = 0.001). As for AI acceptance, H3a (positive effect on perceived effortlessness, β = 0.114, *p* = 0.041) and H3b (negative effect on perceived risk, β = −0.118, *p* = 0.018) are supported, respectively.

Next, we turn to contextual situational factors. Product standardization exerts a significant positive influence on perceived effortlessness (H4a, β = 0.307, *p* < 0.001) and a significant negative influence on perceived risk (H4b, β = −0.185, *p* = 0.001). Platform safeguards positively predict perceived effortlessness (H5a, β = 0.150, *p* = 0.005) and negatively predict perceived risk (H5b, β = −0.394, *p* < 0.001), and the two hypotheses receive support. Social influence is found to significantly enhance perceived effortlessness (H6a, β = 0.135, *p* = 0.017) and significantly reduce perceived risk (H6b, β = −0.167, *p* = 0.015).

Finally, we assess the direct effects of the mediating variables on the outcome construct. Consistent with H7, perceived effortlessness exerts a significant positive effect on individual sellers’ intention to adopt AI chatbots (β = 0.298, *p* < 0.001). In line with H8, perceived risk negatively predicts intention to adopt AI chatbots; this hypothesis is supported (β = −0.430, *p* < 0.001).

In addition to significance testing, we assessed the relative predictive importance of each structural path using *f*^2^ effect size (Hair et al., 2013). The *f*^2^ values evaluate the magnitude of each exogenous construct’s contribution to the variance of an endogenous variable, where values of 0.02, 0.15, and 0.35 represent small, medium, and large effect sizes respectively. As shown in Table 6, no path yields a large effect size, which is reasonable given that the endogenous perceptions are jointly predicted by multiple personal and situational antecedents, and the variance contribution of each single predictor is naturally shared among multiple predictors. Path PB → PE (*f*^2^ = 0.186) and PSA → PR (*f*^2^ = 0.203) show medium effect sizes. PR → IU (*f*^2^ = 0.234) also reaches a medium effect, indicating perceived risk has a stronger predictive weight for adoption intention than perceived effortlessness (PE → IU, *f*^2^ = 0.112). Path PST → PE (*f*^2^ = 0.135) is close to the medium-effect threshold. The remaining hypothesized paths show small but meaningful effect sizes above the 0.02 threshold.

Table 6 presents the standardized path coefficients, significance levels, and *f*^2^ effect sizes for all hypothesized structural paths.

#### 5.2.2. Mediation Analysis

This study further conducts mediation analysis to uncover the underlying indirect mechanisms. Following Zhao et al. (2010), we rely on bootstrapped 95% confidence intervals to judge mediation effects; an indirect effect is significant if the confidence interval excludes zero. Hypotheses H9 and H10 examine the parallel mediating roles of perceived effortlessness and perceived risk. Table 7 summarizes all specific indirect effects. The results reveal that every hypothesized indirect path is significant, given that none of the 95% bootstrap confidence intervals include zero. All sub-hypotheses under H9 (mediation via perceived effortlessness) and H10 (mediation via perceived risk) are therefore supported.

## 6. Discussion

### 6.1. Discussion of Results

This study develops an Integrated UTAUT-TTAT Dual Mediation Model to explore the influencing factors and internal mechanisms of C2C second-hand individual sellers’ adoption of AI chatbots. Drawing on questionnaire data and PLS-SEM analysis, this work reveals that personal factors and situational factors jointly shape individual sellers’ adoption intention through two parallel but opposing perceptual paths: perceived effortlessness and perceived risk. Consistent with the core assumption of this research, individual sellers on C2C second-hand platforms present a typical adoption paradox when facing AI chatbot tools.

Distinct from most prior UTAUT-based studies targeting general consumers or formal B2C merchants (Venkatesh et al., 2012), our sample consists of resource-constrained C2C second-hand independent sellers, whose fragmented work schedules and limited institutional support reshape how personal and situational predictors function in technology decision-making.

In terms of personal factors, perceived busyness is positively associated with perceived effortlessness. While existing time-pressure literature has examined consumer hurriedness (Kim et al., 2019), perceived busyness is rarely treated as a central antecedent within AI-adoption research. For individual sellers burdened by repetitive consulting tasks, AI chatbots function primarily as labour-saving operational tools rather than instruments for performance expansion. Although risk assessment requires substantial time and cognitive resources, busy individual sellers can simultaneously develop favourable effortless perceptions alongside potential risk concerns regarding intelligent services. This finding confirms that perceived busyness operates through both positive and negative perceptual paths, a dynamic less prominent for ordinary consumers or well-resourced corporate individual sellers. As another pivotal personal factor, desire for control exerts dual influences on individual sellers’ perceptual pathways: it positively boosts perceived risk and negatively diminishes perceived effortlessness. C2C second-hand transactions are highly reliant on personalized communication and flexible trading mechanisms. Individual sellers with a strong desire for control tend to worry that standardized automated AI replies may undermine their communication dominance and reduce procedural controllability. Such psychological concerns not only amplify their risk perceptions but also compromise their perceived benefits of AI-driven labour-saving. Prior TTAT-related research often treats control-related needs as peripheral (Carpenter et al., 2019); our findings highlight its critical role for C2C sellers who value interpersonal exchange. AI acceptance constitutes another critical personal trait that positively predicts perceived effortlessness and negatively predicts perceived risk. Individual sellers with positive attitudes toward AI technology are more inclined to recognize the functional advantages of AI chatbots in daily operations, and their inherent trust in intelligent tools effectively mitigates potential risk concerns. This outcome aligns with general technology-acceptance literature.

Regarding situational factors, product standardization, social influence and platform safeguards all exert dual effects on the two mediating constructs. In conventional UTAUT frameworks, product-level attributes are seldom included as direct antecedents of user perceptions. However, within non-standardized second-hand trading contexts, higher product standardization simplifies individual sellers’ information-communication work and reduces transaction disputes, thus improving perceived effortlessness and lowering perceived risk. Rooted in opinions and experience shared by important referents, social influence shapes individual sellers’ cognition of AI chatbots. Positive feedback from various referents helps reduce learning costs and alleviate sellers’ skepticism toward intelligent services. Unlike corporate merchants equipped with brand guarantees, C2C individual sellers heavily depend on platform-provided institutional protection (Pavlou & Gefen, 2004). Sound platform safeguards provide stable transaction rules and dispute-resolution mechanisms, which not only cut down individual sellers’ extra energy consumption but also hedge potential risks brought by intelligent services. The above results fully demonstrate that external situational conditions serve as vital antecedents shaping sellers’ dual perceptions.

Furthermore, the two mediating constructs produce meaningful effects on adoption intention. According to the f^2^ effect-size results, perceived risk carries greater relative predictive importance than perceived effortlessness. This observation extends TTAT applications (Carpenter et al., 2019): for vulnerable individual sellers operating in high-uncertainty second-hand markets, risk-avoidance considerations outweigh efficiency-seeking benefits when forming adoption willingness. Verified parallel mediating effects show that different antecedent variables transmit their influences through distinct perceptual pathways, systematically unpacking the adoption-paradox formation mechanism among C2C second-hand individual sellers.

### 6.2. Theoretical Contributions

This research makes several notable theoretical contributions to the existing literature on AI technology adoption and risk avoidance.

First, this study expands the research context of AI service adoption. Most prior empirical studies on AI chatbot adoption focus on standardized B2C e-commerce scenarios and take consumers or professional merchants as research objects. This paper shifts the research perspective to C2C second-hand platforms and individual sellers, which enriches the empirical evidence of intelligent tool adoption in decentralized, trust-oriented circular e-commerce contexts.

Second, this study extends the application scope of the Unified Theory of Acceptance and Use of Technology (UTAUT) and the Technology Threat Avoidance Theory (TTAT). UTAUT mainly explains users’ positive adoption motivations, while TTAT centers on risk perception and avoidance behaviors. To fully interpret sellers’ attitudes and behavioral intentions toward AI chatbots, we integrate the two theories and establish a dual-path dual mediation model. This framework responds to academic calls for comprehensive analytical frameworks that account for both benefits and potential drawbacks of emerging technologies. It addresses the limitations of single-perspective models and offers a new integrated paradigm for understanding users’ conflicting adoption behaviors.

Third, existing studies on AI adoption predominantly take technical attributes as core antecedents. Moving beyond this dominant perspective, this study incorporates both individual and situational factors into the research model. The empirical results validate the predictive effect of these two categories of factors on users’ dual perceptions. This work complements the antecedent system of technology adoption research and further enhances the theoretical applicability in e-commerce settings.

Fourth, this study enriches the understanding of ambivalent technology adoption mechanisms in resource-constrained individual sellers. Prior AI adoption scholarship mostly examines users with relatively sufficient resources or institutional safeguards, where the tension between perceived benefits and risks is often weak. For C2C second-hand individual sellers with fragmented workload and limited platform protection, the trade-off between effort-saving gains and potential algorithmic risks becomes particularly salient. This work highlights the contextualized adoption paradox of individual sellers and extends the theoretical explanation of competing dual-perception pathways in circular e-commerce.

### 6.3. Practical Implications

Beyond its theoretical contributions, this study yields actionable, stakeholder-specific practical implications for C2C second-hand platform operators, AI chatbot developers, and C2C second-hand individual sellers.

First, for platform operators and AI service developers, it is critical to address individual sellers’ strong demand for workload reduction while accommodating heterogeneous technical capabilities. Platforms may prioritize promoting AI chatbot functions for high-workload sellers through targeted push recommendations. Moreover, developers can design simplified operation modules for less tech-savvy individual sellers to lower the learning burden of intelligent tools and maximize the labour-saving benefits of automated customer consultation.

Second, platform and chatbot designers should mitigate individual sellers’ risk concerns associated with perceived loss of conversational control. Instead of fully automated rigid reply templates, the AI system can reserve adjustable personalized communication settings. For instance, individual sellers can manually override AI-generated replies, customize response tones, and set trigger thresholds for human intervention. Such flexible functions preserve individual sellers’ autonomy and reduce anxiety about losing control of buyer-seller interactions.

Third, platform operators should strengthen institutional safeguard signals and standardized product specification frameworks to reduce perceived risk. Clear, unified templates for second-hand product descriptions help reduce information asymmetry and transaction conflicts. Explicit, visible safeguards—such as platform-mediated dispute arbitration and compensation mechanisms—can serve as tangible risk-mitigation cues. These institutional signals complement AI chatbot services and offset individual sellers’ worries about transaction risks brought by automated conversations.

Fourth, platform communities can cultivate constructive social influence to accelerate the adoption of AI chatbots among hesitant individual sellers. Platforms can host themed sharing sessions led by experienced early adopters, showcasing real operational workflows, cost-saving outcomes and risk-control practices. Disseminating firsthand user experiences helps reduce information gaps and ease unnecessary fears toward AI tools among individual sellers who remain reluctant to adopt.

Finally, for C2C second-hand individual sellers, this study also offers insights into their technology decisions. Individual sellers may evaluate both efficiency gains and potential control-related risks before deploying AI chatbots. They can leverage platform safeguard mechanisms and standardized product information to balance labour-saving benefits and transaction risks when adopting intelligent customer service tools.

### 6.4. Research Limitations and Future Research Directions

Like all empirical studies, this research inevitably has several limitations, which also point out directions for future exploration.

Firstly, this study adopts a cross-sectional questionnaire survey design. The collected data only reflects individual sellers’ perceptions and behavioral intentions at a single time point. Users’ attitudes and adoption behaviors toward AI chatbots may change dynamically with the iteration of platform functions and the accumulation of usage experience. Longitudinal data can be collected in future research to explore the dynamic evolution of adoption mechanisms. In addition, this study only uses quantitative questionnaire data, and future research can adopt mixed-method designs combining interviews or open-ended surveys to supplement contextual qualitative evidence for adoption behaviors.

Secondly, this study only selects perceived effortlessness and perceived risk as two core mediating variables. Other potential mediating factors such as user trust and emotional experience are not included. Follow-up studies can incorporate more psychological constructs to build a more comprehensive multi-mediation model.

Third, this study does not consider moderating variables such as individual sellers’ usage experience and transaction scale. Individual heterogeneity may lead to differences in the influence paths. Future research can add moderating variables to explore the group differences in AI adoption mechanisms.

Fourth, the sample of this study consists of 261 domestic C2C second-hand individual sellers recruited via online questionnaires. All data were obtained through self-reported measures, which may introduce potential common method bias. Although the sample covers diverse seller characteristics (e.g., age, operating years, and monthly transaction frequency), caution should be exercised when generalizing the findings to the whole population of Chinese C2C individual sellers. The applicability of the model to cross-border second-hand platforms and different user groups also needs to be further verified. Subsequent research can adopt stratified sampling based on seller attributes, collect larger samples across multiple second-hand platforms, and expand the sample scope to enhance the generalizability of the research conclusions.

## 7. Conclusions

This study develops and tests an integrated UTAUT-TTAT dual mediation model to elucidate C2C second-hand individual sellers’ attitudinal and behavioral intentions toward AI chatbot adoption. From a behavioral science perspective, this research underscores the importance of balancing benefit perception and risk perception in technology acceptance decision-making. By integrating technology acceptance and threat avoidance theories, the established model reconciles individual sellers’ contradictory psychological perceptions toward intelligent technologies. It provides a balanced theoretical framework for understanding users’ paradoxical adoption behaviors and offers practical implications for the optimization of intelligent service design in C2C second-hand platforms.

## Figures and Tables

**Table 1:** Measurement Constructs and Main Sources.

Construct	Abbreviation	No. of Items	Reference
Perceived Busyness	PB	3	Martin and Park (2003); Kim et al. (2019)
Desire for Control	DC	3	Burger and Cooper (1979)
AI Acceptance	AA	3	Schepman and Rodway (2020)
Product Standardization	PST	3	Self-developed, drawing on Reimann et al. (2010)
Platform Safeguard	PSA	3	Self-developed, drawing on Pavlou and Gefen (2004)
Social Influence	SI	3	Venkatesh et al. (2012); Gansser and Reich (2021)
Perceived Effortlessness	PE	3	Self-developed, drawing on S. M. Sohn (2025)
Perceived Risk	PR	3	Marjerison et al. (2025)
Intention to Use	IU	3	Venkatesh et al. (2012)

**Table 2:** Respondent Profiles (*N* = 261).

Characteristic	Category	No (%)	Characteristic	Category	No (%)
Gender	Male	144 (55.17%)	Monthly transaction frequency	<5 orders	118 (45.21%)
	Female	117 (44.83%)		5–10 orders	86 (32.95%)
Age	18–25	63 (24.14%)		10–20 orders	38 (14.56%)
	25–35	91 (34.87%)		>20 orders	19 (7.28%)
	35–45	77 (29.50%)	AI chatbot usage experience	Never used	141 (54.02%)
	>45	30 (11.49%)		Have used	120 (45.98%)
Operating years	<1 year	27 (10.34%)	AI chatbot usage frequency (among users)	Rarely	46 (38.33%)
	1–3 year	56 (21.46%)		Occasionally	42 (35.00%)
	3–5 year	117 (44.83%)		Frequently	32 (26.67%)
	>5 year	61 (23.37%)			

**Table 3:** Convergent Validity and Internal Consistency Reliability.

Construct	Items	Loading	Item Reliability	Cronbach’s α	CR	AVE
PB	PB1	0.913	0.834	0.863	0.916	0.784
	PB2	0.879	0.773			
	PB3	0.864	0.746			
DC	DC1	0.899	0.808	0.881	0.926	0.808
	DC2	0.912	0.832			
	DC3	0.885	0.783			
AA	AA1	0.941	0.885	0.898	0.936	0.830
	AA2	0.923	0.852			
	AA3	0.866	0.750			
PST	PST1	0.919	0.845	0.887	0.930	0.816
	PST2	0.914	0.835			
	PST3	0.876	0.767			
PSA	PSA1	0.935	0.874	0.918	0.948	0.859
	PSA2	0.923	0.852			
	PSA3	0.922	0.850			
SI	SI1	0.918	0.843	0.868	0.918	0.789
	SI2	0.918	0.843			
	SI3	0.825	0.681			
PE	PE1	0.830	0.689	0.775	0.869	0.689
	PE2	0.833	0.694			
	PE3	0.827	0.684			
PR	PR1	0.934	0.872	0.888	0.930	0.817
	PR2	0.901	0.812			
	PR3	0.875	0.766			
IU	IU1	0.970	0.941	0.943	0.963	0.898
	IU2	0.944	0.891			
	IU3	0.928	0.861			

**Table 4:** Inter-Construct Correlations and Summary Statistics.

	M	SD	1	2	3	4	5	6	7	8	9
1. PB	4.055	1.390	0.885								
2. DC	3.476	1.362	−0.042	0.899							
3. AA	4.015	1.408	0.110	−0.017	0.911						
4. PST	3.971	1.395	0.344 ***	0.018	0.194 **	0.903					
5. PSA	3.851	1.496	0.156 *	−0.065	0.204 **	0.182 **	0.927				
6. SI	4.198	1.402	0.036	0.070	−0.023	0.121 *	−0.342 ***	0.888			
7. PE	4.052	0.901	0.503 ***	−0.136	0.242 ***	0.491 ***	0.246 ***	0.122	0.830		
8. PR	3.907	1.313	−0.360 ***	0.193 **	−0.256 ***	−0.369 ***	−0.439 ***	−0.048	−0.474 ***	0.904	
9. IU	3.922	1.583	0.346 ***	−0.094	0.121	0.352 ***	0.259 ***	0.071	0.498 ***	−0.567 ***	0.948
Year	2.812	0.911	0.058	−0.076	−0.092	−0.058	−0.050	−0.047	−0.005	0.046	−0.022

Note. The diagonal elements represent the square root of AVE. *** *p* < 0.001, ** *p* < 0.01, * *p* < 0.05. Year = operating years of individual sellers, serving as the marker variable for common method bias testing.

**Table 5:** Heterotrait-Monotrait (HTMT) Ratios of Correlations.

	1	2	3	4	5	6	7	8	9
1. PB									
2. DC	0.050								
3. AA	0.130	0.030							
4. PST	0.395	0.062	0.214						
5. PSA	0.171	0.073	0.223	0.201					
6. SI	0.057	0.079	0.039	0.139	0.386				
7. PE	0.612	0.165	0.285	0.591	0.291	0.147			
8. PR	0.413	0.216	0.281	0.413	0.485	0.063	0.572		
9. IU	0.380	0.102	0.131	0.385	0.273	0.080	0.578	0.618	

Note. Values represent HTMT ratios. Discriminant validity is established when the HTMT value is below 0.85.

**Table 6:** Structural Model Results.

Hypotheses	Path	β	t-Value	*p*-Value	*f* ^2^	Conclusion
H1a	PB → PE	0.351	6.967	<0.001	0.186	Supported
H1b	PB → PR	−0.208	3.963	<0.001	0.062	Supported
H2a	DC → PE	−0.125	2.341	0.019	0.027	Supported
H2b	DC → PR	0.172	3.195	0.001	0.048	Supported
H3a	AA → PE	0.114	2.078	0.038	0.021	Supported
H3b	AA → PR	−0.118	2.386	0.018	0.021	Supported
H4a	PST → PE	0.307	5.619	<0.001	0.135	Supported
H4b	PST → PR	−0.185	3.283	0.001	0.046	Supported
H5a	PSA → PE	0.150	2.821	0.005	0.031	Supported
H5b	PSA → PR	−0.394	6.053	<0.001	0.203	Supported
H6a	SI → PE	0.135	2.394	0.017	0.027	Supported
H6b	SI → PR	−0.167	2.427	0.015	0.039	Supported
H7	PE → IU	0.298	5.423	<0.001	0.112	Supported
H8	PR → IU	−0.430	7.533	<0.001	0.234	Supported

**Table 7:** Mediation hypotheses and specific indirect effects.

Hypotheses	Path	β	t-Value	95% CI	Conclusion
H9a	PB → PE → IU	0.105	4.273	[0.061, 0.156]	Supported
H9b	DC → PE → IU	−0.037	2.107	[−0.077, −0.007]	Supported
H9c	AA → PE → IU	0.034	1.968	[0.004, 0.072]	Supported
H9d	PST → PE → IU	0.092	3.600	[0.049, 0.150]	Supported
H9e	PSA → PE → IU	0.045	2.498	[0.015, 0.086]	Supported
H9f	SI → PE → IU	0.040	2.163	[0.006, 0.080]	Supported
H10a	PB → PR → IU	0.090	3.376	[0.043, 0.149]	Supported
H10b	DC → PR → IU	−0.074	2.970	[−0.126, −0.029]	Supported
H10c	AA → PR → IU	0.051	2.323	[0.011, 0.093]	Supported
H10d	PST → PR → IU	0.080	2.914	[0.031, 0.138]	Supported
H10e	PSA → PR → IU	0.170	4.567	[0.104, 0.250]	Supported
H10f	SI → PR → IU	0.072	2.249	[0.012, 0.135]	Supported

## Data Availability

The anonymized data supporting the findings of this study is available from the corresponding author upon reasonable request.

## Supplementary Materials

The following supporting information can be downloaded at: https://www.mdpi.com/article/10.3390/bs16091670/s1, Table S1. Measurement Items and Item Sources.
