# Supplementary material for: AI Chatbot Service Adoption Among C2C Second-Hand Sellers: An Integrated UTAUT-TTAT Framework
Source: Behav Sci (Basel). 2026 Sep 17;16(9):1670. doi: 10.3390/bs16091670 (PMC13603910; doi:10.3390/bs16091670)
Supplement: Supplementary file 1 [file behavsci-16-01670-s001.zip › behavsci-4552948-supplementary.pdf]

Supplementary Materials

Table S1. Measurement Items and Item Sources.

Construct	Items	Reference
Perceived Busyness (PB)	PB1-I have a great number of tasks to handle every day.<br>PB2-I often feel pressed for time.<br>PB3-My store receives a large volume of daily inquiries and orders.	Martin & Park (2003); Kim et al. (2019)
Desire for Control (DC)	DC1-I prefer to take full charge of all customer service work in my store.<br>DC2-I tend to make decisions on my own rather than relying on tools.<br>DC3-I like to have full control over my work procedures.	Burger & Cooper (1979)
AI Acceptance (AA)	AA1-I generally trust artificial intelligence technologies.<br>AA2-I am open to try new AI tools emerging in daily consumption.<br>AA3-In general, I hold positive views toward the development of AI.	Schepman & Rodway (2020)
Product Standardization (PST)	PST1-The brand, model and specifications of my products are completed in a uniform and standard manner.<br>PST2-I use consistent standards to describe product conditions and defects.<br>PST3-The titles and description templates of my products are largely unified.	Self-developed, drawing on Reimann et al. (2010)
Platform Safeguard (PSA)	PSA1-The platform provides complete guidelines for using AI customer service.<br>PSA2-The platform guarantees transaction security during the use of AI customer service.<br>PSA3-The platform has compensation and liability mechanisms for errors made by AI customer service.	Self-developed, drawing on Pavlou & Gefen (2004)
Social Influence (SI)	SI1-People who influence my behavior think that I should use AI chatbot service.<br>SI2-People who are important to me think that I should use AI chatbot service.<br>SI3-People whose opinion I value prefer to use AI chatbot service.	Venkatesh et al. (2012); Gansser & Reich (2021)
Perceived Effortlessness (PE)	PE1-Using the platform's AI chatbot service saves me a lot of time on replying to inquiries.<br>PE2-AI chatbot service reduces my workload of manual customer service.<br>PE3-With AI chatbot service, I do not need to repeatedly explain product information to buyers.	Self-developed, drawing on Sohn (2025)
Perceived Risk (PR)	PR1-I worry that the AI chatbot service will make mistakes when introducing products.<br>PR2-I am concerned that inappropriate replies from AI will dissatisfy buyers.<br>PR3-I fear that AI errors will lead to transaction disputes.	Marjerison et al. (2025)
Intention to Use (IU)	IU1–I intend to use the platform's AI chatbot service in the future.<br>IU2–I will always try to use the platform's AI chatbot service in my transactions.<br>IU3–I plan to use the platform's AI chatbot service frequently.	Venkatesh et al. (2012)
